# The genome sequence of the Green Oak Leaf Roller, *Tortrix viridana* (Linnaeus, 1758)

**DOI:** 10.12688/wellcomeopenres.21568.1

**Published:** 2024-05-15

**Authors:** Douglas Boyes, Liam M. Crowley, Lucy M. Morley, University of Oxford and Wytham Woods Genome Acquisition Lab, Darwin Tree of Life Barcoding collective, Wellcome Sanger Institute Tree of Life Management, Samples and Laboratory team, Wellcome Sanger Institute Scientific Operations: Sequencing Operations, Wellcome Sanger Institute Tree of Life Core Informatics team, Tree of Life Core Informatics collective, Darwin Tree of Life Consortium

**Affiliations:** 1 UK Centre for Ecology & Hydrology, Wallingford, England, UK; 2 Department of Biology, University of Oxford, Oxford, England, UK

**Keywords:** Tortrix viridana, green oak leaf roller, genome sequence, chromosomal, Lepidoptera

## Abstract

We present a genome assembly from a female *Tortrix viridana* (the Green Oak Leaf Roller; Arthropoda; Insecta; Lepidoptera; Tortricidae). The genome sequence is 456.6 megabases in span. Most of the assembly is scaffolded into 31 chromosomal pseudomolecules, including the Z and W sex chromosomes. The mitochondrial genome has also been assembled and is 16.43 kilobases in length.

## Species taxonomy

Eukaryota; Opisthokonta; Metazoa; Eumetazoa; Bilateria; Protostomia; Ecdysozoa; Panarthropoda; Arthropoda; Mandibulata; Pancrustacea; Hexapoda; Insecta; Dicondylia; Pterygota; Neoptera; Endopterygota; Amphiesmenoptera; Lepidoptera; Glossata; Neolepidoptera; Heteroneura; Ditrysia; Apoditrysia; Tortricoidea; Tortricidae; Tortricinae; Tortricini; *Tortrix*; *Tortrix viridana* (Linnaeus, 1758) (NCBI:txid311328).

## Background

The Green Oak Leaf Roller or Green Oak Tortrix, *Tortrix viridana*, is a moth of the Tortricidae family ( Sterling *et al.*, 2023). It is common and widespread across much of Britain ( Davis, 2012). It is widely distributed across Europe and east to Asia Minor ( GBIF Secretariat, 2024). *T. viridana* has a wingspan of 17–24 mm and is a very distinctive micromoth, being the only UK tortricid species with unmarked bright green forewings and head ( Sterling *et al.*, 2023). However, it may appear similar to the macro-moth the Cream-bordered Green Pea ( *Earias chlorana*; Nolidae). *Earias chlorana* may be distinguished by a broader white line along the costa and by the fact that it rests with the wings held tent-like, rather than flat as with *T. viridana* ( Sterling *et al.*, 2023). *T. viridana* hindwings are light grey.

The adults flight period occurs from mid-May to July and they are attracted to light ( Sterling *et al.*, 2023). The species is univoltine and females lay eggs in pairs on the bark of twigs. Larvae hatch in the following spring, between April and June, to feed on flushing buds during the earlier instars and then on the fresh expanding leaves as later instars ( Du Merle, 1999). Larvae are greyish olive green in colour, with a black or dark brown head, dark pinacula and black thoracic legs. It is narrowly oligophagous, hatching and feeding predominantly on oaks ( *Quercus* spp.) and *T. viridana* is an abundant Lepidopteran species in oak woodlands ( Du Merle, 1999). As the common name suggests, the larvae roll or fold host leaves to form a feeding tube that they feed within ( Sterling *et al.,* 2023).

*T. viridana* is considered a pest of oaks in Europe, as it undergoes population cycles with periodic outbreaks leading to heavy defoliation that may reduce wood and acorn production and make trees more vulnerable to further pest attacks, e.g. oak powdery mildew ( *Erysiphe* ( *Microsphaera*) *alphitoides*) ( Schroeder & Degen, 2008a; Szabóky & Csóka, 2010). Therefore, research has aimed to untangle the influential factors on *T. viridana’s* cyclical dynamics, including the role of host tree phenology, competition (e.g. with *Operophtera brumata*), dispersal and mating behaviour, and population genetics across space, to which this full reference genome will further contribute ( Hunter, 1990; Hunter, 1998; Schroeder & Degen, 2008a; Schroeder & Degen, 2008b; Simchuk *et al.*, 1999).

## Genome sequence report

**Figure 1.**
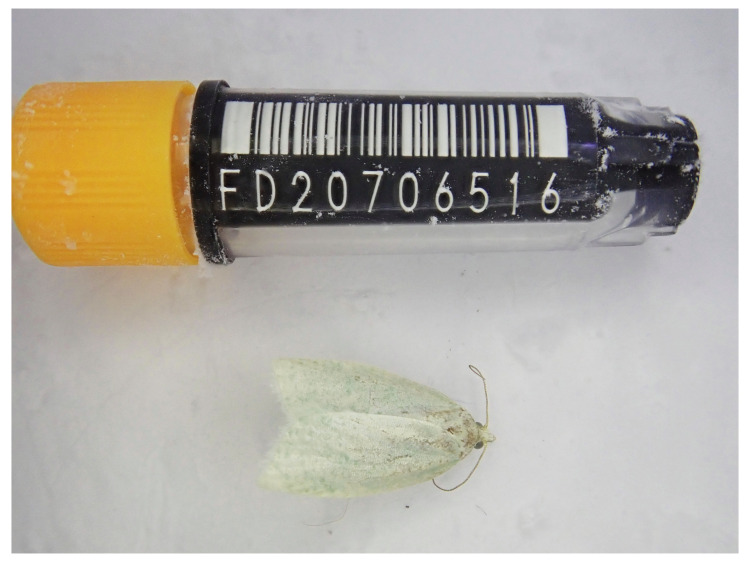
Photograph of the *Tortrix viridana* (ilTorViri5) specimen used for genome sequencing.

The genome was sequenced from a female *Tortrix viridana* ( Figure 1) collected from Wytham Woods, Wytham Woods, UK (51.77, –1.34). A total of 60-fold coverage in Pacific Biosciences single-molecule HiFi long reads was generated. Primary assembly contigs were scaffolded with chromosome conformation Hi-C data. Manual assembly curation corrected 3 missing joins or mis-joins and removed 1 haplotypic duplication, reducing the scaffold number by 4.44%.

**Figure 2.**
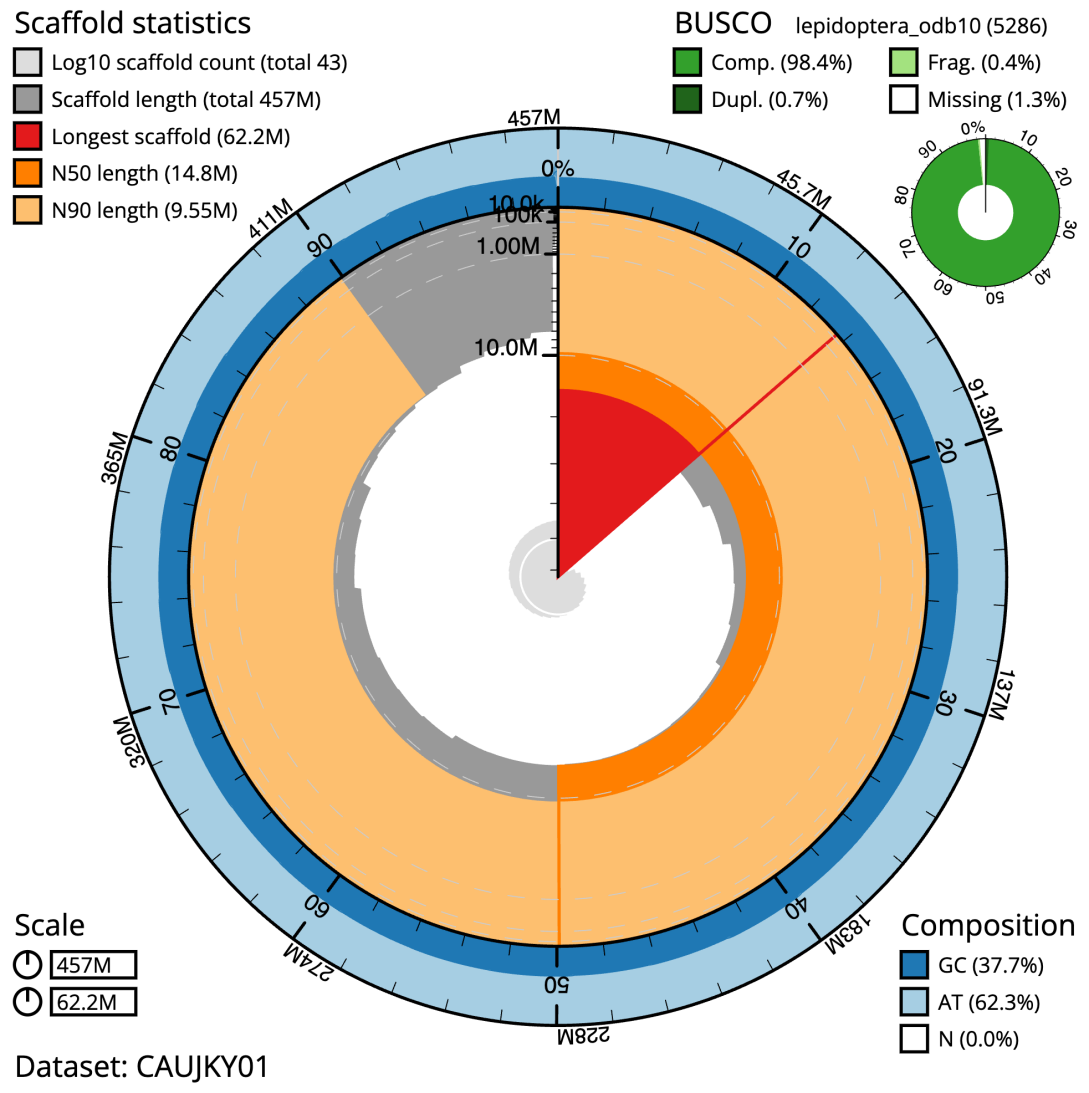
Genome assembly of *Tortrix viridana*, ilTorViri5.1: metrics. The BlobToolKit snail plot shows N50 metrics and BUSCO gene completeness. The main plot is divided into 1,000 size-ordered bins around the circumference with each bin representing 0.1% of the 456,586,594 bp assembly. The distribution of scaffold lengths is shown in dark grey with the plot radius scaled to the longest scaffold present in the assembly (62,234,340 bp, shown in red). Orange and pale-orange arcs show the N50 and N90 scaffold lengths (14,768,249 and 9,547,436 bp), respectively. The pale grey spiral shows the cumulative scaffold count on a log scale with white scale lines showing successive orders of magnitude. The blue and pale-blue area around the outside of the plot shows the distribution of GC, AT and N percentages in the same bins as the inner plot. A summary of complete, fragmented, duplicated and missing BUSCO genes in the lepidoptera_odb10 set is shown in the top right. An interactive version of this figure is available at https://blobtoolkit.genomehubs.org/view/CAUJKY01/dataset/CAUJKY01/snail.

**Figure 3.**
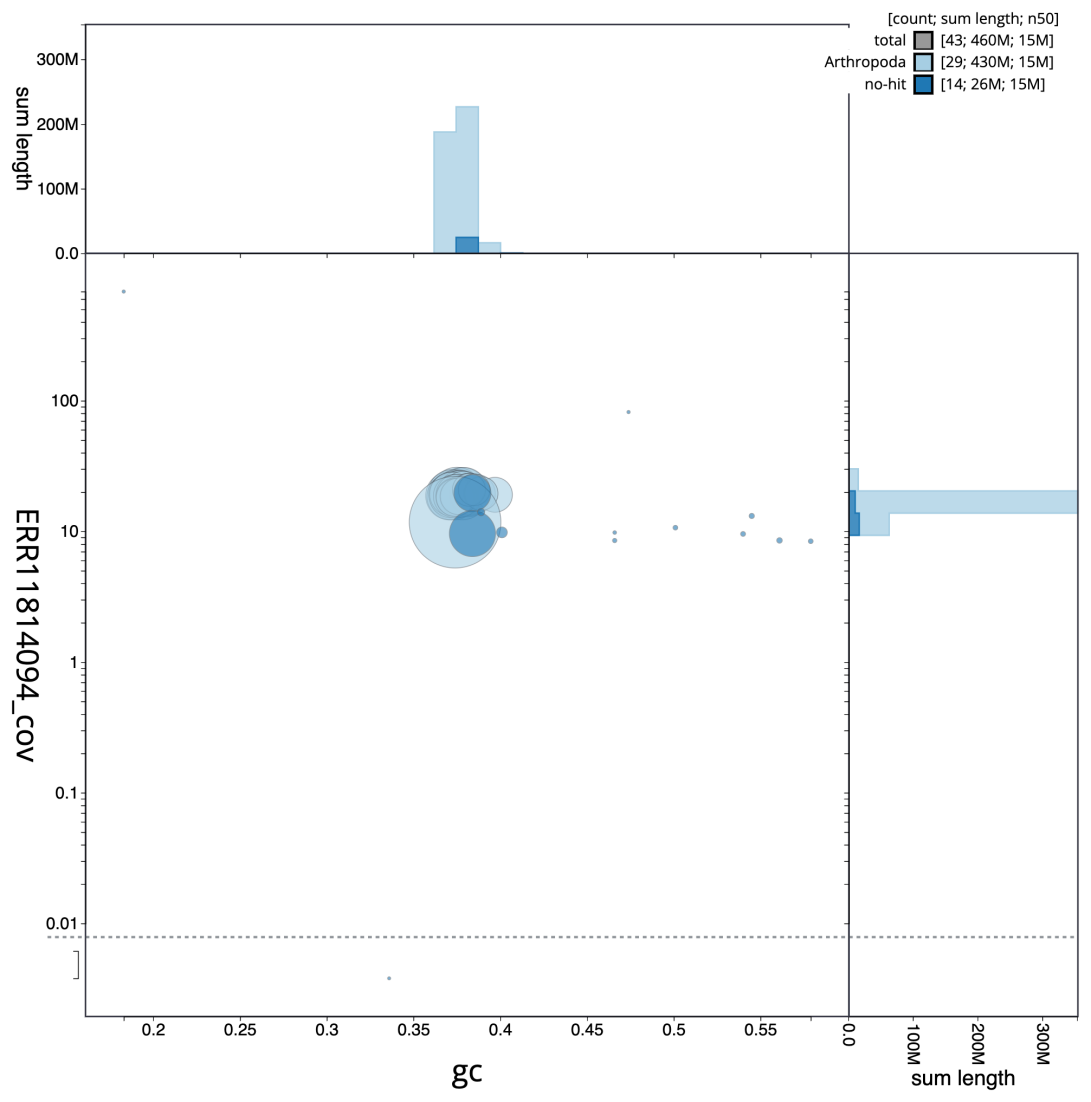
Genome assembly of *Tortrix viridana*, ilTorViri5.1: BlobToolKit GC-coverage plot. Sequences are coloured by phylum. Circles are sized in proportion to sequence length. Histograms show the distribution of sequence length sum along each axis. An interactive version of this figure is available at https://blobtoolkit.genomehubs.org/view/CAUJKY01/dataset/CAUJKY01/blob.

**Figure 4.**
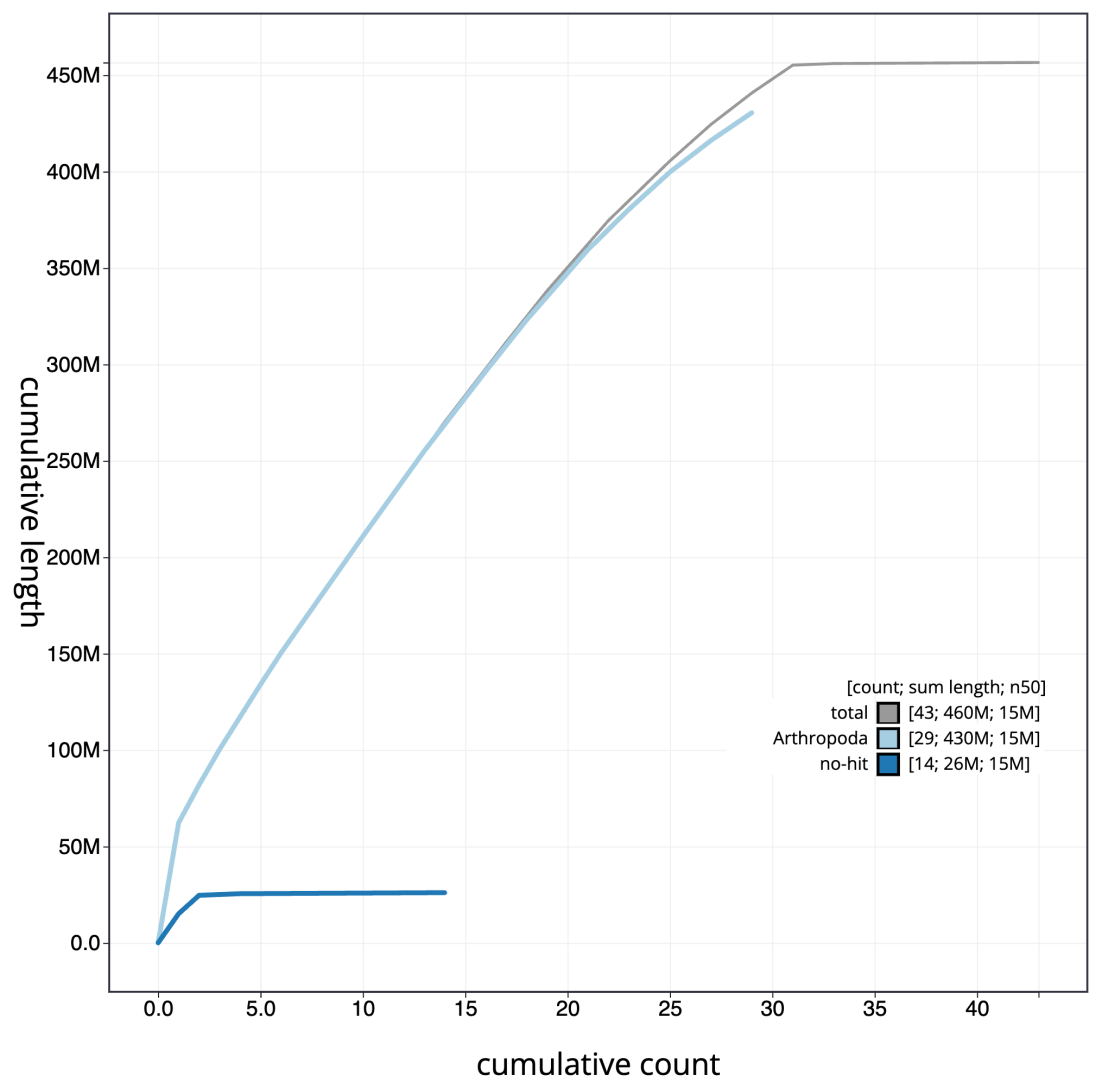
Genome assembly of *Tortrix viridana*, ilTorViri5.1: BlobToolKit cumulative sequence plot. The grey line shows cumulative length for all sequences. Coloured lines show cumulative lengths of sequences assigned to each phylum using the buscogenes taxrule. An interactive version of this figure is available at https://blobtoolkit.genomehubs.org/view/CAUJKY01/dataset/CAUJKY01/cumulative.

**Figure 5.**
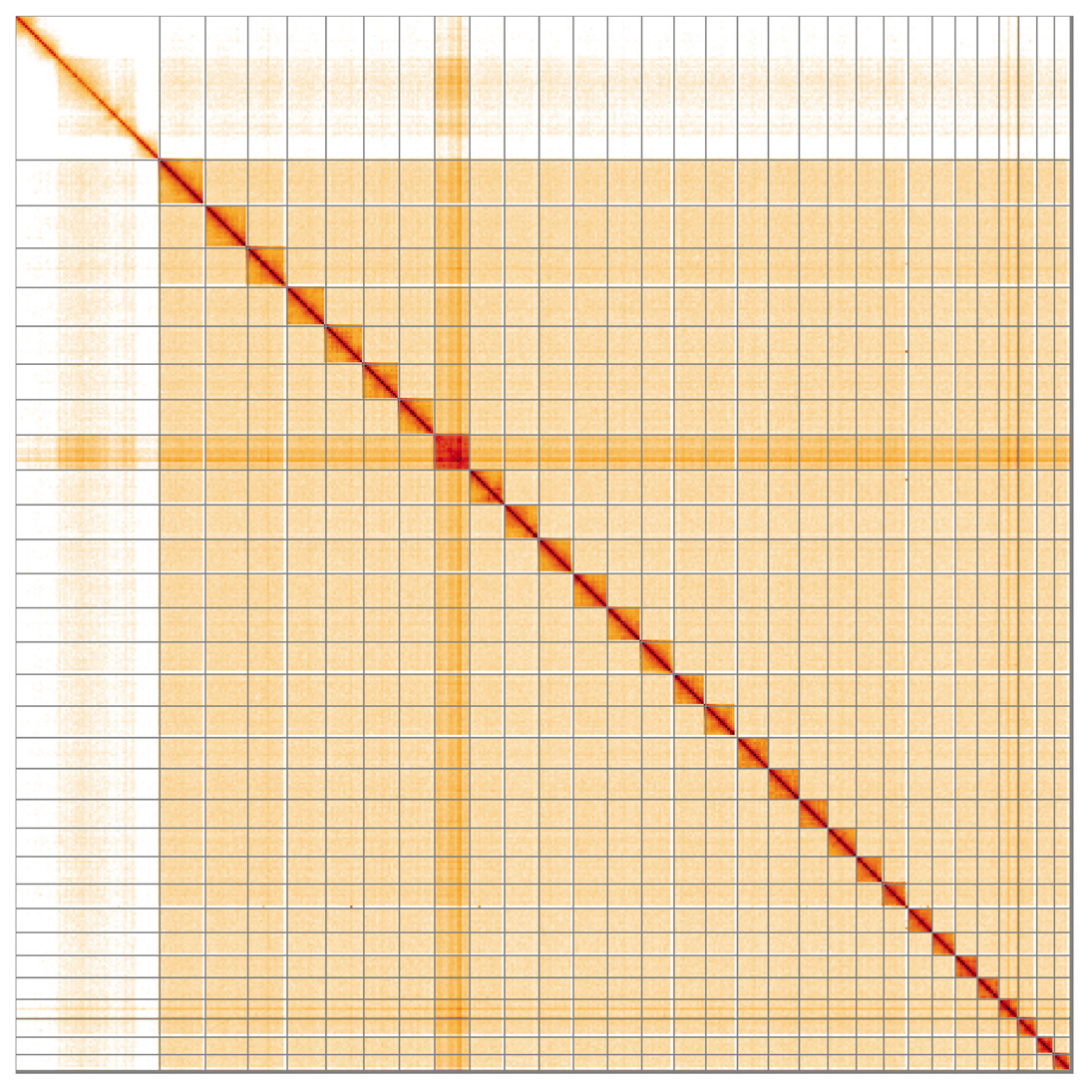
Genome assembly of *Tortrix viridana*, ilTorViri5.1: Hi-C contact map of the ilTorViri5.1 assembly, visualised using HiGlass. Chromosomes are shown in order of size from left to right and top to bottom. An interactive version of this figure may be viewed at https://genome-note-higlass.tol.sanger.ac.uk/l/?d=YTZURUETToenilpT27Mfeg.

The final assembly has a total length of 456.6 Mb in 42 sequence scaffolds with a scaffold N50 of 14.8 Mb ( Table 1). The snail plot in Figure 2 provides a summary of the assembly statistics, while the distribution of assembly scaffolds on GC proportion and coverage is shown in Figure 3. The cumulative assembly plot in Figure 4 shows curves for subsets of scaffolds assigned to different phyla. Most (99.88%) of the assembly sequence was assigned to 31 chromosomal-level scaffolds, representing 29 autosomes and the Z and W sex chromosomes. Chromosome-scale scaffolds confirmed by the Hi-C data are named in order of size ( Figure 5; Table 2). While not fully phased, the assembly deposited is of one haplotype. Contigs corresponding to the second haplotype have also been deposited. The mitochondrial genome was also assembled and can be found as a contig within the multifasta file of the genome submission.

**Table 1.** Genome data for *Tortrix viridana*, ilTorViri5.1.

Project accession data		
Assembly identifier	ilTorViri5.1	
Species	*Tortrix viridana*	
Specimen	ilTorViri5	
NCBI taxonomy ID	311328	
BioProject	PRJEB64712	
BioSample ID	SAMEA10978932	
Isolate information	ilTorViri5, female: whole organism (DNA and Hi-C sequencing)	
Assembly metrics *		*Benchmark*
Consensus quality (QV)	64.4	*≥ 50*
*k*-mer completeness	100.0%	*≥ 95%*
BUSCO **	C:98.4%[S:97.7%,D:0.7%], F:0.4%,M:1.3%,n:5,286	*C ≥ 95%*
Percentage of assembly mapped to chromosomes	99.88%	*≥ 95%*
Sex chromosomes	ZW	*localised homologous pairs*
Organelles	Mitochondrial genome: 16.43 kb	*complete single alleles*
Raw data accessions		
PacificBiosciences SEQUEL II	ERR11809137	
Hi-C Illumina	ERR11814102	
Genome assembly		
Assembly accession	GCA_963241965.1	
*Accession of alternate haplotype*	GCA_963243755.1	
Span (Mb)	456.6	
Number of contigs	88	
Contig N50 length (Mb)	10.1	
Number of scaffolds	42	
Scaffold N50 length (Mb)	14.8	
Longest scaffold (Mb)	62.23	

* Assembly metric benchmarks are adapted from column VGP-2020 of “Table 1: Proposed standards and metrics for defining genome assembly quality” from Rhie *et al.* (2021). ** BUSCO scores based on the lepidoptera_odb10 BUSCO set using version 5.3.2. C = complete [S = single copy, D = duplicated], F = fragmented, M = missing, n = number of orthologues in comparison. A full set of BUSCO scores is available at https://blobtoolkit.genomehubs.org/view/CAUJKY01/dataset/CAUJKY01/busco.

**Table 2.** Chromosomal pseudomolecules in the genome assembly of *Tortrix viridana*, ilTorViri5.

INSDC accession	Chromosome	Length (Mb)	GC%
OY725172.1	1	19.7	38.0
OY725173.1	2	18.37	37.5
OY725174.1	3	16.95	38.0
OY725175.1	4	16.72	37.0
OY725176.1	5	16.34	37.5
OY725178.1	6	15.34	37.0
OY725179.1	7	15.23	37.5
OY725180.1	8	15.0	37.5
OY725181.1	9	14.96	37.0
OY725182.1	10	14.77	37.5
OY725183.1	11	14.77	37.5
OY725184.1	12	14.73	37.0
OY725185.1	13	13.96	37.5
OY725186.1	14	13.73	37.0
OY725187.1	15	13.61	38.0
OY725188.1	16	13.34	37.5
OY725189.1	17	13.33	38.0
OY725190.1	18	12.36	37.5
OY725191.1	19	12.25	38.5
OY725192.1	20	11.87	37.5
OY725193.1	21	10.56	38.5
OY725194.1	22	10.35	37.5
OY725195.1	23	9.95	37.5
OY725196.1	24	9.55	38.5
OY725197.1	25	9.34	37.5
OY725198.1	26	8.45	39.5
OY725199.1	27	7.9	39.0
OY725200.1	28	7.5	38.0
OY725201.1	29	6.93	38.5
OY725177.1	W	15.12	38.5
OY725171.1	Z	62.23	37.5
OY725202.1	MT	0.02	18.5

The estimated Quality Value (QV) of the final assembly is 64.4 with *k*-mer completeness of 100.0%, and the assembly has a BUSCO v5.3.2 completeness of 98.4% (single = 97.7%, duplicated = 0.7%), using the lepidoptera_odb10 reference set ( *n* = 5,286).

Metadata for specimens, barcode results, spectra estimates, sequencing runs, contaminants and pre-curation assembly statistics are given at https://links.tol.sanger.ac.uk/species/311328.

## Methods

### Sample acquisition and nucleic acid extraction

A female *Tortrix viridana* (specimen ID Ox001663, ToLID ilTorViri5) was netted in Wytham Woods, Oxfordshire (biological vice-county Berkshire), UK (latitude 51.77, longitude –1.34) on 2021-07-17. The specimen was collected by Douglas Boyes (University of Oxford) and identified by Liam Crowley (University of Oxford) and preserved on dry ice.

The workflow for high molecular weight (HMW) DNA extraction at the Wellcome Sanger Institute (WSI) includes a sequence of core procedures: sample preparation; sample homogenisation, DNA extraction, fragmentation, and clean-up. In sample preparation, the ilTorViri5 sample was weighed and dissected on dry ice ( Jay *et al.*, 2023). Tissue from the whole organism was homogenised using a PowerMasher II tissue disruptor ( Denton *et al.*, 2023a). HMW DNA was extracted using the Automated MagAttract v2 protocol ( Oatley *et al.*, 2023a). DNA was sheared into an average fragment size of 12–20 kb in a Megaruptor 3 system ( Bates *et al.*, 2023). Sheared DNA was purified by solid-phase reversible immobilisation ( Oatley *et al.*, 2023b): in brief, the method employs a 1.8X ratio of AMPure PB beads to sample to eliminate shorter fragments and concentrate the DNA. The concentration of the sheared and purified DNA was assessed using a Nanodrop spectrophotometer and Qubit Fluorometer and Qubit dsDNA High Sensitivity Assay kit. Fragment size distribution was evaluated by running the sample on the FemtoPulse system.

Protocols developed by the WSI Tree of Life laboratory are publicly available on protocols.io ( Denton *et al.*, 2023b).

### Sequencing

Pacific Biosciences HiFi circular consensus DNA sequencing libraries were constructed according to the manufacturers’ instructions. DNA sequencing was performed by the Scientific Operations core at the WSI on a Pacific Biosciences SEQUEL II instrument. Hi-C data were also generated from remaining tissue of ilTorViri5 using the Arima2 kit and sequenced on the Illumina NovaSeq 6000 instrument.

### Genome assembly, curation and evaluation

Assembly was carried out with Hifiasm ( Cheng *et al.*, 2021) and haplotypic duplication was identified and removed with purge_dups ( Guan *et al.*, 2020). The assembly was then scaffolded with Hi-C data ( Rao *et al.*, 2014) using YaHS ( Zhou *et al.*, 2023). The assembly was checked for contamination and corrected using the gEVAL system ( Chow *et al.*, 2016) as described previously ( Howe *et al.*, 2021). Manual curation was performed using gEVAL, HiGlass ( Kerpedjiev *et al.*, 2018) and PretextView ( Harry, 2022). The mitochondrial genome was assembled using MitoHiFi ( Uliano-Silva *et al.*, 2023), which runs MitoFinder ( Allio *et al.*, 2020) or MITOS ( Bernt *et al.*, 2013) and uses these annotations to select the final mitochondrial contig and to ensure the general quality of the sequence.

A Hi-C map for the final assembly was produced using bwa-mem2 ( Vasimuddin *et al.*, 2019) in the Cooler file format ( Abdennur & Mirny, 2020). To assess the assembly metrics, the *k*-mer completeness and QV consensus quality values were calculated in Merqury ( Rhie *et al.*, 2020). This work was done using Nextflow ( Di Tommaso *et al.*, 2017) DSL2 pipelines “sanger-tol/readmapping” ( Surana *et al.*, 2023a) and “sanger-tol/genomenote” ( Surana *et al.*, 2023b). The genome was analysed within the BlobToolKit environment ( Challis *et al.*, 2020) and BUSCO scores ( Manni *et al.*, 2021; Simão *et al.*, 2015) were calculated.

Table 3 contains a list of relevant software tool versions and sources.

**Table 3.** Software tools: versions and sources.

Software tool	Version	Source
BlobToolKit	4.2.1	https://github.com/blobtoolkit/blobtoolkit
BUSCO	5.3.2	https://gitlab.com/ezlab/busco
gEVAL	N/A	https://geval.org.uk/
Hifiasm	0.19.5-r587	https://github.com/chhylp123/hifiasm
HiGlass	1.11.6	https://github.com/higlass/higlass
Merqury	MerquryFK	https://github.com/thegenemyers/MERQURY.FK
MitoHiFi	3	https://github.com/marcelauliano/MitoHiFi
PretextView	0.2	https://github.com/wtsi-hpag/PretextView
purge_dups	1.2.5	https://github.com/dfguan/purge_dups
sanger-tol/genomenote	v1.0	https://github.com/sanger-tol/genomenote
sanger-tol/readmapping	1.1.0	https://github.com/sanger-tol/readmapping/tree/1.1.0
YaHS	1.2a.2	https://github.com/c-zhou/yahs

### Wellcome Sanger Institute – Legal and Governance

The materials that have contributed to this genome note have been supplied by a Darwin Tree of Life Partner. The submission of materials by a Darwin Tree of Life Partner is subject to the **‘Darwin Tree of Life Project Sampling Code of Practice’**, which can be found in full on the Darwin Tree of Life website here. By agreeing with and signing up to the Sampling Code of Practice, the Darwin Tree of Life Partner agrees they will meet the legal and ethical requirements and standards set out within this document in respect of all samples acquired for, and supplied to, the Darwin Tree of Life Project.

Further, the Wellcome Sanger Institute employs a process whereby due diligence is carried out proportionate to the nature of the materials themselves, and the circumstances under which they have been/are to be collected and provided for use. The purpose of this is to address and mitigate any potential legal and/or ethical implications of receipt and use of the materials as part of the research project, and to ensure that in doing so we align with best practice wherever possible. The overarching areas of consideration are:

•     Ethical review of provenance and sourcing of the material

•     Legality of collection, transfer and use (national and international) 

Each transfer of samples is further undertaken according to a Research Collaboration Agreement or Material Transfer Agreement entered into by the Darwin Tree of Life Partner, Genome Research Limited (operating as the Wellcome Sanger Institute), and in some circumstances other Darwin Tree of Life collaborators.

## Data Availability

European Nucleotide Archive: *Tortrix viridana*. Accession number PRJEB64712; https://identifiers.org/ena.embl/PRJEB64712 ( Wellcome Sanger Institute, 2023). The genome sequence is released openly for reuse. The *Tortrix viridana* genome sequencing initiative is part of the Darwin Tree of Life (DToL) project. All raw sequence data and the assembly have been deposited in INSDC databases. The genome will be annotated using available RNA-Seq data and presented through the Ensembl pipeline at the European Bioinformatics Institute. Raw data and assembly accession identifiers are reported in Table 1.

## Author information

Members of the University of Oxford and Wytham Woods Genome Acquisition Lab are listed here: https://doi.org/10.5281/zenodo.7125292.

Members of the Darwin Tree of Life Barcoding collective are listed here: https://doi.org/10.5281/zenodo.4893703

Members of the Wellcome Sanger Institute Tree of Life Management, Samples and Laboratory team are listed here: https://doi.org/10.5281/zenodo.10066175.

Members of Wellcome Sanger Institute Scientific Operations: Sequencing Operations are listed here: https://doi.org/10.5281/zenodo.10043364.

Members of the Wellcome Sanger Institute Tree of Life Core Informatics team are listed here: https://doi.org/10.5281/zenodo.10066637.

Members of the Tree of Life Core Informatics collective are listed here: https://doi.org/10.5281/zenodo.5013541.

Members of the Darwin Tree of Life Consortium are listed here: https://doi.org/10.5281/zenodo.4783558.
