# Supplementary material for: The genome sequence of the Green Oak Leaf Roller, Tortrix viridana (Linnaeus, 1758)
Source: Wellcome Open Res. 2024 May 15;9:260. [Version 1] doi: 10.12688/wellcomeopenres.21568.1 (PMC11467647; doi:10.12688/wellcomeopenres.21568.1)
Supplement: Peer review history

# Reviewer report: Reviewer response for version 1

**DOI:** 10.21956/wellcomeopenres.23840.r102248

**Published:** 2024-10-10

**Authors:** Thayná Bisson Ferraz Lopes

**Affiliations:** 1 Universidade Estadual de Londrina, Londrina, State of Paraná, Brazil

This data note presents a high-quality assembly of the genome of the Green Oak Leaf Roller, *Tortrix viridana*.

 The background section encompasses all pertinent information regarding this species. The methodologies used for sample acquisition, nucleic acid extraction, sequencing, and genome assembly were adequate and well described. The figures and assembly statistics provide valuable insights into the quality and validation of the assembly.

 This data will serve as an invaluable resource for researchers studying moth genomics and ecology in future investigations.

Are sufficient details of methods and materials provided to allow replication by others?

Yes

Is the rationale for creating the dataset(s) clearly described?

Yes

Are the datasets clearly presented in a useable and accessible format?

Yes

Are the protocols appropriate and is the work technically sound?

Yes

Reviewer Expertise:

Bioinformatics and molecular genetics of insects

I confirm that I have read this submission and believe that I have an appropriate level of expertise to confirm that it is of an acceptable scientific standard.

# Reviewer report: Reviewer response for version 1

**DOI:** 10.21956/wellcomeopenres.23840.r101525

**Published:** 2024-10-10

**Authors:** Kathy Darragh

**Affiliations:** 1 Indiana University Bloomington, Bloomington, Indiana, USA

The authors describe their genome assembly of a female *Tortrix viridana *moth (green oak leaf roller). Background on the ecology and natural history of the moth is provided. The data collection, sequencing, and assembly methods are all clearly described and appropriate. This provides useful data for public use through the tree of life project.

Are sufficient details of methods and materials provided to allow replication by others?

Yes

Is the rationale for creating the dataset(s) clearly described?

Yes

Are the datasets clearly presented in a useable and accessible format?

Yes

Are the protocols appropriate and is the work technically sound?

Yes

Reviewer Expertise:

Evolutionary biology

I confirm that I have read this submission and believe that I have an appropriate level of expertise to confirm that it is of an acceptable scientific standard.

# Reviewer report: Reviewer response for version 1

**DOI:** 10.21956/wellcomeopenres.23840.r97129

**Published:** 2024-09-17

**Authors:** Ningning Wu

**Affiliations:** 1 China Agricultural University College of Plant Protection, Beijing, Beijing, China

The rationale for generating the dataset is clearly. The genome of *Tortrix viridana* is presented as valuable for understanding the species’ cyclical population dynamics and its role as a pest in European oak forests. The dataset is contextualized within research on Lepidopteran species and pest management, with potential contributions to future studies on population genetics, pest control, and forest ecosystem health.

 However, the rationale could be enhanced by directly highlighting specific scientific questions or hypotheses this dataset addresses, to emphasize its broader relevance. The protocols are appropriate for genome assembly. The inclusion of multiple steps such as manual curation and contamination checks further ensures the technical rigor of the work.

 Additionally, the use of standardized tools like Hifiasm, purge_dups, and BUSCO analysis provides confidence in the accuracy and completeness of the assembly. Overall, the technical work appears sound. The manuscript provides thorough descriptions of the methods, including the DNA extraction workflow, sequencing, assembly, and curation steps. Protocols for high molecular weight DNA extraction, shearing, and library preparation are sufficiently detailed, with relevant references to publicly available protocols.io entries, making replication feasible.

 The datasets are presented in a clear and accessible manner. The assembly statistics, BUSCO scores, and figures are informative and provide useful insights into the quality of the assembly. The inclusion of metadata and external links to barcode results, spectra estimates, and assembly statistics further enhances the usability of the data.

Are sufficient details of methods and materials provided to allow replication by others?

Yes

Is the rationale for creating the dataset(s) clearly described?

Yes

Are the datasets clearly presented in a useable and accessible format?

Yes

Are the protocols appropriate and is the work technically sound?

Yes

Reviewer Expertise:

Genome evolution

I confirm that I have read this submission and believe that I have an appropriate level of expertise to confirm that it is of an acceptable scientific standard.
